# Immunoadjuvanted influenza vaccine immunogenicity in children with type 1 diabetes over two consecutive seasons

**DOI:** 10.3389/fimmu.2025.1597619

**Published:** 2025-10-08

**Authors:** Mikhail Petrovich Kostinov, Maria Alexandrovna Kvasova, Alla Anatolievna Tarasova, Anna Vlasenko, Elena Vladimirovna Kolbasina, Darya Alexandrovna Bydanova, Aristitsa Kostinova, Valentina Polishchuk, Isabella Abramovna Khrapunova, Marina Loktionova, Andrey Viktorovich Linok, Yulia Alekseevna Dagil, Elena Petrovna Foshina

**Affiliations:** ^1^ Ivan Mikhailovich I.M. Sechenov First Moscow State Medical University, Moscow, Russia; ^2^ Ilya Ilyich I.I. Mechnikov Research Institute of Vaccines and Sera (RAS), Moscow, Russia; ^3^ Federal State Budgetary Educational Institution of Higher Education (Privolzhsky Research Medical University) of the Ministry of Health of the Russian Federation, Nizhny Novgorod, Russia; ^4^ Digital Technologies and Platforms, Moscow, Russia; ^5^ Nizhny Novgorod Regional Pediatric Clinical Hospital, Nizhny Novgorod, Russia; ^6^ Central Research Institute of Epidemiology (CRIE), Moscow, Russia; ^7^ Institute of Immunology, Moscow, Russia

**Keywords:** children, type 1 diabetes mellitus, influenza vaccine, Immunogenicity, antibodies to influenza virus

## Abstract

Patients with diabetes mellitus face a significantly higher risk of severe influenza and its complications. The purpose of this study was to investigate the immunogenicity of the trivalent immunoadjuvanted subunit influenza vaccine in children with type 1 diabetes (T1D) over two consecutive seasons.

**Methods:**

A prospective non-randomized study during 2 epidemic seasons included 146 children with T1D at the age of 12.0 (9.0-14.0) years; the main group consisted of 81 patients vaccinated against influenza, the control group included 65 unvaccinated children. Antibody (Ab) levels to influenza viruses were evaluated using the hemagglutination inhibition assay before vaccination, one month and 12 months after vaccination.

**Results:**

Over two seasons, vaccinated children with T1D demonstrated a significant increase in Ab against all three vaccine strains 1 month post-vaccination, irrespective of their initial specific Ab levels. Differences in the persistence of antibodies 12 months post-vaccination were observed between children initially seronegative for A/H1N1 and A/H3N2 strains, who exhibited lower antibodies levels and fold increases, and those initially seropositive. Vaccinated seropositive children experienced significant post-vaccination Ab increases, surpassing levels in initially seronegative patients. Regardless of the epidemiological season, vaccination significantly increased the chance of achieving a seroprotective Ab level within one month for the A/H1N1 strain by 4.7 [2.9-9.7] (χ²M-H = 16.4, p < 0.001), for the A/H3N2 strain by 15.8 [5.9-41.4] (χ²M-H = 44.0, p < 0.001), and for strain B by 14.8 [6.5-33.6] (χ²M-H = 46.2, p < 0.001). Twelve months post-vaccination, Ab persistence was highest for the B strain, with levels 7.2 [3.2–16] times higher than in unvaccinated children, regardless of the season. Persistence of antibodies to the A/H1N1 strain was season-dependent (lower in the 2015-2016 season) and 2.5 [1.3-5] times higher than in unvaccinated children (χ²M-H = 6.5, p = 0.01). Antibodies persistence to the A/H3N2 strain did not differ significantly between vaccinated and unvaccinated groups (1.0 [0.5-2.3], χ²M-H = 0.02, p = 0.89).

**Conclusion:**

Administration of the trivalent immunoadjuvanted subunit influenza vaccine in children with T1D resulted in the formation of postvaccination Ab, meeting the Committee for Proprietary Medicinal Products (CPMP) immunogenicity criteria regardless of vaccination history.

## Introduction

The global rise in the incidence and prevalence of type 1 diabetes (T1D) among children and adolescents has become a significant public health concern over recent decades (1–4). Infectious agents, primarily influenza viruses (5), are becoming increasingly important in the onset and decompensation of diabetes (6, 7). In patients with diabetes, the frequency of hospitalizations for influenza or pneumonia is significantly higher than in people without diabetes (OR 5.08 [3.45; 7.50]; p <0.00001), including hospitalizations in intensive care units, and they experience more severe outcomes compared to individuals without diabetes (7, 8).

Vaccination is a cornerstone of influenza prevention and its associated complications. The World Health Organization (WHO) recommends achieving at least 75% vaccination coverage among vulnerable populations to effectively prevent influenza (9). Influenza vaccination is particularly recommended for all patients with diabetes (10, 11). It positively impacts the course of the underlying disease, reducing hospitalizations for acute diabetes complications—such as ketoacidosis, hypoglycemia, and coma - by 11% (12), and reducing the risk of death by 17% during the influenza season (13).

Several recent studies have shown that influenza vaccination is effective in patients with diabetes, but some researchers consider it insufficiently effective (14–16). The lack of effectiveness is believed to be due to insulin resistance leading to a series of immune reactions that exacerbate the inflammatory condition, which leads to hyperglycemia. In addition, congenital and adaptive defects of the immune response, including dysfunction of neutrophils, macrophages, T cells, increase susceptibility to invading pathogens in patients with diabetes (17) Therefore, the search for novel approaches to the treatment and prevention of infectious diseases, in particular more effective vaccination strategies against influenza, is relevant.

In studies comparing high-dose and standard-dose trivalent influenza vaccines (14) and quadrivalent vaccine (18) in adults over 60 years of age with chronic conditions, higher relative efficacy, immunogenicity and seroprotection were demonstrated with high doses of vaccine. However, no separate analysis was performed for patients with diagnosed diabetes.

Data on the results of influenza vaccine use in children with T1D are limited. In search of a vaccination strategy that would safely provide sustainable protection for people from any population group with high efficiency, attention should be paid to the immunoadjuvanted vaccine against influenza, which was used in children with T1D (19). It was shown that in the early stages of vaccination, the level of seroprotection (antibodies (Ab) titer > 1:40) to the A/H1N1 strain was 84%, the seroconversion level (4-fold increase in the level of Ab) was 66%, the seroconversion factor (the average multiplicity of increase in the titer of Ab and 95% confidence interval) was 20.6 (10.4–30.9); to the A/H3N2 strain: 98%, 41% and 9.9 (3.2-16.6), in the influenza B virus: 77%, 49% and 7.7 (4.0-11.4), respectively. One year after vaccination, the proportion of children with protective antibody levels (>1:40) was as follows: influenza A/H1N1 virus: 74% (32/43 children), influenza A/H3N2 virus: 88% (38/43 children), influenza B virus: 58% (25/43 children). Thus, while the ability to synthesize protective levels of influenza virus Ab in children with T1D was verified, further research is required to understand the development of post-vaccination immunity, particularly its dependence on initial antibody levels and the longevity of their persistence.

The aim of this study was to investigate the immunogenicity of the trivalent immunoadjuvanted subunit influenza vaccine in children with T1D over two consecutive seasons.

## Materials

### Description of study groups

A prospective non-randomized study in parallel groups included the main group of 81 T1D patients vaccinated against influenza, and the control group of 65 children with T1D whose parents declined vaccination against influenza during the study seasons. The distribution of patients by vaccination season is presented in **Table 1**.

**Table 1:** The distribution of patients by vaccination season.

Groups	Season		Total
	2014-2015	2015-2016	
Vaccinated against influenza	46	35	81
Control group	33	32	65

The vaccination status of participants was confirmed by reviewing of electronic immunization program “Vaccine prophylaxis” and the child`s individual development card records. The diabetes was diagnosed according to the WHO diagnostic criteria. The WHO diagnostic criteria for diabetes include: a fasting plasma glucose level of 7.0 mmol/L (126 mg/dL) or higher, or a 2-hour plasma glucose level of 11.1 mmol/L (200 mg/dL) or higher during an oral glucose tolerance test, or a glycated hemoglobin (HbA1c) level of 6.5% or higher (20).

#### Inclusion criteria

children aged 3 to 17 years with type 1 diabetes;

and written informed consent obtained from parents for study participation.

#### Non-inclusion criteria

ketonuria (acetone in the urine);

acute infectious and somatic diseases or exacerbations of chronic diseases in the period of less than 1 month before vaccination;

presence of organic lesions of the central nervous system, oncological diseases, HIV infection;

allergic reactions to chicken egg protein or other components of the influenza vaccine.

prior influenza vaccination within six months before study participation;

administration of other vaccines within one month before influenza vaccination.

#### Exclusion criteria

non-compliance with the study protocol;

refusal to continue participation.

### Ethical review

The study was approved by the local ethical council of the I.I. Mechnikov Research Institute of Vaccines and Serums, protocol No. 2/2014 dated 10.03.2014.

Children were enrolled in the study from the endocrinology department and the consultative and diagnostic center of the State Budgetary Healthcare Institution “Nizhny Novgorod Regional Children’s Clinical Hospital” (NRCCH, Russia) during outpatient consultations.

The characteristics of the sample in general and separately of the study groups are presented in **Table 2**. The age of the patients was 12.0 (9.0-14.0) years. The gender distribution was approximately equal, with 53% girls and 47% boys. The study groups were generally comparable in terms of gender, age, duration of the T1D and most of the analyzed characteristics. However, notable differences were identified: in the group of vaccinated patients, the level of glycated hemoglobin was higher (8.9 (7.6; 10.5) % versus 7.9 (7.2; 9.0) %, p = 0.006). In the control group, no children had been vaccinated against influenza in previous seasons, whereas 56 (69%) of the vaccinated group had received influenza vaccination in the preceding season (95% CI: 58–78%).

**Table 2:** Characteristics of the sample and study groups.

Parameters	Total (N = 146)	Vaccinated (N = 81)	Unvaccinated (N = 65)	Between groups
Age, years	12.0 (9.0-14.0)	12.0 (9.0; 14.5)	11.0 (8.5; 13.5)	p = 0.24
Glycated hemoglobin, %	8.4 (7.4-9.8)	8.9 (7.6; 10.5)	7.9 (7.2; 9.0)	p = 0.006
Cholesterol, mmol/l	3.9 (3.5-4.5)	3.9 (3.5; 4.7)	3.9 (3.4; 4.3)	p = 0.14
Creatinine, μmol/l	53 (46-52)	56 (48-68)	52 (45-59)	p = 0.11
Glucose, mmol/l	7.5 (5.6-9.3)	7.7 (5.8; 9.4)	7.3 (5.6; 8.9)	p = 0.59
Diabetes mellitus experience, years	3 (2-6)	4 (2-6)	3 (2-5)	p=0.36
Antibodies to GAD	11 (8%) [4-13]	6 (7%) [3-15]	5 (8%) [3-17]	p = 0.95
Gender (proportion of men)	69 (47%) [39-55]	40 (49%) [39-60]	29 (45%) [33-57]	p=0.57
Over 5 years of diabetes experience	52 (36%) [28-44]	31 (38%) [28-49]	21 (32%) [22-44]	p = 0.46
Previously vaccinated	56 (38%) [31-46]	56 (69%) [58-78]	0 (0%) [0-6]	p<0.001
Season 2014-2015	79 (54%) [46-62]	46 (57%) [46-67]	33 (51%) [39-63]	p = 0.47
Season 2015-2016	67 (46%) [38-54]	35 (43%) [33-54]	32 (49%) [37-61]	
Seronegative to A/H1N1	84 (58%) [49-65]	47 (58%) [47-68]	37 (57%) [45-68]	p=0.89
Seronegative to A/H3N2	74 (51%) [43-59]	46 (57%) [46-67]	28 (43%) [32-55]	p = 0.10
Seronegative to B	94 (64%) [56-72]	47 (58%) [47-68]	47 (72%) [60-82]	p = 0.07
Polyneuropathy	79 (54%) [46-62]	43 (53%) [42-64]	36 (55%) [43-67]	p=0.78
Retinopathy	3 (2%) [1-6]	2 (2%) [1-9]	1 (2%) [0-8]	p=1.00
Small anomalies of heart development	97 (66%) [58-74]	56 (69%) [58-78]	41 (63%) [51-74]	p = 0.44
Biliary tract dysfunction	73 (50%) [42-58]	41 (51%) [40-61]	32 (49%) [37-61]	p = 0.87
Vegetative-vascular dystonia	11 (8%) [4-13]	6 (7%) [3-15]	5 (8%) [3-17]	p = 0.94
Autoimmune thyroiditis	15 (10%) [6-16]	10(12%)[7-21]	5 (8%) [3-17]	p=0.36
Dysmetabolic nephropathy	22(15%)[10-22]	13(16%)[10-26]	9(14%)[7-24]	p=0.71
Myopia	11 (8%) [4-13]	6 (7%) [3-15]	5 (8%) [3-17]	p = 0.95

^1^ – Mann-Whitney criterion was applied in case of quantitative data and χ^2^ criterion for nominal data (Fisher, if there are cells in the table with expected frequencies ≤5%)

Among the concomitant diseases, the most common were minor cardiac anomalies (in 66% of patients), biliary tract dysfunction (in 50% of patients), dysmetabolic nephropathy (in 15% of all patients). The prevalence of concomitant diseases did not differ significantly between the study groups.

### Description of medical intervention

According to the National Immunization Schedule of the Russian Federation, children with T1D were vaccinated with the trivalent immunoadjuvanted subunit influenza vaccine Grippol Plus (NPO Petrovax Pharm, LLC, Russia). Each dose of the vaccine contains 5 μg of hemagglutinin of each of the current epidemic strains of the influenza virus (A/H1N1, A/H3N2 and B) cultivated on chicken embryos (Abbott Biologicals B.V., Netherlands), and 500 μg of water-soluble immunoadjuvant polyoxidonium (NPO Petrovax Pharm LLC, Russia). Polyoxidonium (azoximer bromide) is registered in Russia as an immunotropic drug and has been widely used in children and adults for over 20 years (21, 22).

Epidemic strains recommended by WHO for 2014–2015 northern hemisphere vaccines were influenza type A antigen allantoic subtype (H1N1) A/California/7/2009pdm09-like (NYMC X-181), influenza type A antigen allantoic subtype (H3N2) A/Texas/50/2012-like (NYMC X-223A), influenza type B antigen allantoic B/Massachusetts/2/2012-like (NYMC BX-51B). Recommended reference viruses for 2015/2016 vaccines were allantoic influenza type A antigen A/Bolivia/559/2013 (H1N1)-like to strain A/California/07/2009 (H1N1) pdm09, allantoic influenza type A antigen subtype (H3N2) A/Switzerland/9713293/2013; allantoic influenza type B virus antigen B/Phuket/3073/2013 (23). The strain composition of the vaccines used in the study was in line with the WHO recommendations for the Northern Hemisphere and the EU decision on influenza vaccine composition for the 2014–2015 and 2015–2016 seasons. The vaccine is used according to the National Immunization Schedule in children from 6 months of age, for all age categories in adults, pregnant women, and individuals with chronic diseases (24–29).

## Methods

### Determination of antibody levels

Determination of Ab levels to strains of influenza viruses was carried out according to the hemagglutination inhibition (HI) method using influenza diagnostics of the following strains for the season 2014/2015: A/H1N1/California/07/09-like, A/H3N2/Texas/50/12-like, B/Massachusetts/2/12- like; for the season 2015/2016: study of Ab to strains A/H1N1Bolivia/559/2013-like, A/H3N2/Switzerland/9713293/2013; B/Phuket/3073/2013. (LLC “Enterprise for the production of diagnostic drugs”, Russia).

To remove non-specific hemagglutination inhibitors, the test sera were treated with receptor-destroying enzyme (RDE; Denka Seiken, Tokyo, Japan). 50 µl of the test serum was mixed with 150 µl of RDE and incubated at 37 °C overnight (19 ± 1 h), followed by 30-min inactivation at 56 °C and dilution to 1:10 with phosphate buffer. The HI reaction was performed with 0.5% chicken erythrocytes and 4 units of hemagglutination antigens. Antigens for the HI reaction were provided by the Smorodintsev Research Institute of Influenza (WHO National Influenza Center, St. Petersburg).

### Biochemical analyzes

At the time of enrollment, children with T1D underwent baseline biochemical analyses, which included the following measurements: the level of glycosylated hemoglobin (HbA1c) was measured using an automatic DS5 Glycomat analyzer (BioChemMac, Russia) with low pressure cation exchange chromatography; blood glucose was determined using individual glucometers, and acetone in urine was measured using Dorui test strips for professional use (Dorui Industrial Company, Ltd., China).

### Antibody analysis

The analysis of patient samples was carried out in the laboratory of vaccine prevention and immunotherapy of allergic diseases at the institute using certified equipment of the Center for Collective Use of the Federal State Budgetary Scientific Institution I.I. Mechnikov Research Institute of Vaccines and Serums. To remove non-specific hemagglutination inhibitors, the test sera were treated with receptor-destroying enzyme (RDE; Denka Seiken, Tokyo, Japan). 50 µl of the test serum was mixed with 150 µl of RDE and incubated at 37 °C overnight (19 ± 1 h), followed by 30-min inactivation at 56 °C and dilution to 1:10 with phosphate buffer. The HI reaction was performed with 0.5% chicken erythrocytes and 4 units of hemagglutination antigens. Antigens for the HI reaction were provided by the Smorodintsev Research Institute of Influenza (WHO National Influenza Center, St. Petersburg).

Blood samples were collected to assess Ab levels to influenza strains in both groups at three time points: at the time of enrollment, one month after vaccination, twelve months after vaccination. A titer of 1:40 or higher was considered seroprotective, while patients with titers below 1:40 were classified as seronegative The immunogenicity of the influenza vaccine was evaluated according to the efficacy criteria for adult patients aged 18–60 years established by the Committee for Proprietary Medicinal Products (CPMP/BWP/214/96), The criteria included: 1) seroprotection level: the proportion of vaccinated individuals in which the titer of hemagglutinin-inhibiting antibodies exceeded 1:40 by the 21st day after vaccination (target: >70%); 2) seroconversion level or immunological activity of the vaccine: the ratio of the number of vaccinated individuals in which the titer of hemagglutinin-inhibiting antibodies increased more than 4-fold compared to the initial level, and in which the level was not lower than 1:40 on the 21st day (target > 40%); 3) seroconversion factor (geometric mean fold rise, GMFR): an increase in the geometric mean level of hemagglutinin-inhibiting Ab on the 21st day compared to baseline, expressed as a fold increase (target: >2.5).

A vaccine is considered immunogenic if it meets at least one of these three criteria for each strain. Therefore, the determination of the seroprotection level, as well as the seroconversion level or seroconversion factor, allows us to evaluate the immunological effectiveness of the vaccine. If the vaccine meets the above immunogenicity criteria, the best epidemiological effect can be expected, especially if the seasonal circulating influenza viruses match the strain composition of the vaccine.

The study of patient samples was conducted in the laboratory of vaccination prevention and immunotherapy of allergic diseases of the institute using certified equipment of the shared use center of the Federal State Budgetary Scientific Institution “I.I. Mechnikov Research Institute of Vaccines and Serums”.

### Statistical analysis methods and sample size calculation

The sample size calculation was based on immunogenicity criteria, particularly a seroprotection level of at least 70%. In a pilot sample of 30 patients, the proportion of patients with a protective level of Ab (≥40) was assessed; it was 27% to the A/H1N1 strain, 34% to the A/H3N2 strain and 19% to the B strain. With an expected post-vaccination increase to at least 70%, the calculated effect sizes were 0.88 for the A/H1N1 strain, 0.74 for the A/H3N2 strain and 1.08 for the B strain. Using the pwr package and the pwr.2p.test function based on the specified information for a study power of 80%, the minimum size of each study group was calculated, which was 20 for the A/H1N1 strain, 29 for the A/H3N2 strain and 14 for the B strain (30). The total number of study groups (**Table 1**) was 81 people in the vaccinated group and 65 patients in the control group, of which 79 patients were recruited in the 2014–2015 season. (N = 46 in the vaccinated group and N = 33 in the control group) and 67 in the 2015–2016 season. (N = 35 in the vaccinated group and N = 32 in the control group).

The Shapiro-Wilk test was used to evaluate the normality of distribution of characteristics. Significant deviations from normality were identified. Descriptive statistics of the Ab level are represented by the geometric mean titer (GMT) and its 95% confidence interval: GMT [95% CI]. Descriptive statistics of the multiplicity increase (seroconversion factor) is also represented as the geometric mean and its 95% confidence interval: GMFR [95% CI]. Descriptive statistics of other quantitative characteristics are presented by the median and interquartile range Med (Q1-Q3). The change in quantitative indicators (differences between baseline and specific time points) is represented by the median of the corresponding series of differences and its 95% confidence interval: Δ [95% CI].

To compare two unrelated samples by quantitative indicator, the Mann-Whitney test was employed. Longitudinal analysis of characteristic changes and inter-group comparisons were performed by constructing a robust linear mixed effects model (RLMEM) (31). The statistical significance of the model coefficients was determined using the Satterthwaite approximation for degrees of freedom (32). *Post hoc* comparisons (between groups at different time points and within groups over time) were performed by constructing the relevant contrasts using the calculated model and the emmeans package, with the Benjamini-Hochberg correction applied (33). The construction of the model for the Ab level was carried out on the data after the logarithmic transformation, for the remaining characteristics, modeling was carried out on the initial data. As fixed factors in the analysis of the formation of post-vaccination immunity, the following were determined: time after vaccination (1 month, 12 months), vaccination season (2014-2015/2015-2016), gender (male/female), age group (≥12 years/<12 years), duration of T1D (≥5 years/<5 years), prior influenza vaccination (yes/no), the initial level of Ab to the analyzed strain (seropositive/seronegative). Individual patients were assigned as random factors. Marginal R2 and conditional R2 were calculated to assess the model quality (34).

For qualitative variables, absolute and relative frequencies (in percentages) were calculated. For relative indicators, 95% CIs were determined using the Wilson method. Two groups were compared by qualitative nominal parameters using cross-tabulation analysis with the χ² test or Fisher’s exact test when expected cell frequencies were ≤5%. For paired qualitative data (e.g., pre- and post-vaccination), the McNemar test was used. To assess the strength of the relationship between vaccination and the level of seroprotection, the odds ratio was calculated, showing how many times higher the chance of achieving a seroprotective level in the vaccinated compared with the non-vaccinated. The Mantel-Henszel method was used to generalize the odds ratio (by seasons), and the Cochran-Mantel-Henszel test was applied; the homogeneity of the odds ratio was checked by the Breslow-Day test.

Statistical significance was set at p ≤ 0.05. For all multiple comparisons, the Benjamini-Hochberg correction was applied. Analyses and visualizations were performed using GraphPad Prism (v.9.3.0 License GPS-1963924) and the R statistical environment (v.3.6, License GNU GPL2).

## Results

### Antibodies to A/H1N1 strain

The analysis of post-vaccination immunity (changes of the Ab level over time) was carried out taking into account gender, age of the patient, duration of T1D, and previous vaccination history. A robust linear mixed-effects model was used to analyze Ab level against the A/H1N1 strain. It was revealed that the increase in the Ab level, adjusted for interfering factors relative to the initial level, differed significantly (p <0.001) based on vaccination status (**Table 3**, all cases). It should be mentioned that the changes of the level of antibodies over time to the A/H1N1 strain a year after vaccination in the group of vaccinated patients depended on the season (p = 0.002), and in general, the level of antibodies depended on its baseline value (p <0.001).

**Table 3:** Robust mixed-effects model parameters for the Ab level separately for each strain.

Factors		A/H1N1			A/H3N2			B/Yamagata lineage		
		B ± SE	T	P	B ± SE	T	P	B ± SE	T	P
All cases (N = 81 in the vaccinated group, N = 65 in the control group)										
Main effects	Season (2015–2016 vs 2014-2015)	-0.09 ± 0.11	0.8	p = 0.40	-0.32 ± 0.10	3.1	p = 0.002	0.13 ± 0.09	1.5	p = 0.15
	1-month period (vs baseline)	0.00 ± 0.08	0.1	p=0.96	-0.20 ± 0.09	2.3	p = 0.02	-0.07 ± 0.08	-0.8	p = 0.42
	12 months period (vs baseline)	-0.06 ± 0.08	0.8	p = 0.42	-0.08 ± 0.09	0.9	p=0.37	-0.14 ± 0.08	-1.6	p = 0.11
	Gender (women vs men)	-0.14 ± 0.13	1.1	p = 0.28	0.09 ± 0.10	0.9	p=0.36	0.02 ± 0.07	0.3	p=0.74
	Age ≥ 12 years (vs <12 years)	0.03 ± 0.15	0.2	p = 0.83	0.09 ± 0.11	0.8	p = 0.41	0.33 ± 0.08	4.1	p<0.001
	Experience of diabetes ≥ 5 years (vs <5 years)	0.10 ± 0.06	1.7	p = 0.10	-0.01 ± 0.05	0.2	p = 0.82	0.13 ± 0.08	1.6	p = 0.12
	Previously vaccinated	0.11 ± 0.09	1.2	p = 0.22	-0.02 ± 0.07	0.3	p = 0.73	-0.06 ± 0.05	1.1	p = 0.26
	Initially seronegative	-0.72 ± 0.06	11.8	p<0.001	-0.55 ± 0.06	9.8	p<0.001	-0.54 ± 0.04	15.3	p<0.001
Iteration	Vaccination × 1 month	0.78 ± 0.10	7.9	p<0.001	0.65 ± 0.11	5.8	p<0.001	0.58 ± 0.11	5.2	p<0.001
	Vaccination × 1 month × season	-0.32 ± 0.14	1.9	p = 0.07	0.13 ± 0.16	0.8	p = 0.44	0.06 ± 0.16	0.4	p = 0.70
	Vaccination × 12 months	0.54 ± 0.10	5.5	p<0.001	0.33 ± 0.11	3.0	p = 0.003	0.36 ± 0.11	3.3	p = 0.001
	Vaccination × 12 month × season	-0.46 ± 0.14	3.2	p = 0.002	-0.17 ± 0.16	1.0	p = 0.31	-0.03 ± 0.16	0.2	p = 0.87
R^2^ conditional (R^2c^), R^2^ marginal (R^2m^)		R^2c^=0.77, R^2m^=0.53			R^2c^=0.68, R^2m^=0.57			R^2c^=0.58, R^2m^=0.58		
Only seronegative (SN-)										
Main	N in the vaccinated group	N = 47			N = 46			N = 47		
	N in the control group	N = 37			N = 28			N = 47		
	Season 2015-2016 (vs 2014-2015)	0.03 ± 0.16	0.2	p = 0.83	0.27 ± 0.16	1.7	p = 0.09	0.16 ± 0.10	1.6	p = 0.11
	1-month period (vs baseline)	0.05 ± 0.11	0.5	p = 0.64	0.10 ± 0.20	0.5	p = 0.62	-0.01 ± 0.09	0.1	p = 0.95
	12 months period (vs baseline)	0.04 ± 0.11	0.3	p = 0.73	0.11 ± 0.19	0.6	p = 0.60	-0.07 ± 0.07	0.8	p = 0.44
	Gender women (vs men)	-0.21 ± 0.20	1.1	p = 0.27	-0.06 ± 0.11	0.5	p = 0.61	-0.11 ± 0.09	1.2	p = 0.22
	Age ≥ 12 years (vs <12 years)	0.40 ± 0.21	1.8	p = 0.07	0.18 ± 0.11	1.6	p = 0.11	0.12 ± 0.08	1.1	p = 0.28
	Experience of diabetes ≥ 5 years (vs <5 years)	0.17 ± 0.10	1.8	p = 0.08	-0.01 ± 0.05	0.1	p=0.89	0.18 ± 0.10	1.9	p = 0.07
	Previously vaccinated	0.27 ± 0.13	1.7	p = 0.09	0.01 ± 0.07	0.1	p = 0.98	-0.11 ± 0.06	1.7	p = 0.10
	Initially seronegative	0.02 ± 0.11	0.2	p = 0.83	0.03 ± 0.06	0.4	p = 0.68	0.02 ± 0.05	0.3	p=0.74
Iteration	Vaccination × 1 month	0.97 ± 0.13	7.3	p <0.001	1.05 ± 0.23	4.5	p<0.001	0.69 ± 0.12	5.5	p<0.001
	Vaccination × 1 month × season	-0.54 ± 0.20	-2.8	p = 0.007	-0.39 ± 0.27	1.5	p = 0.14	0.17 ± 0.20	0.9	p = 0.39
	Vaccination × 12 months	0.65 ± 0.13	4.9	p<0.001	0.75 ± 0.23	3.2	p = 0.002	0.41 ± 0.12	3.3	p = 0.001
	Vaccination × 12 month × season	-0.55 ± 0.20	-2.8	p = 0.006	-0.72 ± 0.27	2.7	p = 0.008	-0.04 ± 0.20	0.2	p = 0.86
R^2^ conditional (R^2c^), R^2^ marginal (R^2m^)		R^2c^=0.75, R^2m^=0.40			R^2c^=0.56, R^2m^=0.56			R^2c^=0.51, R^2m^=0.51		

**Figure 1:**
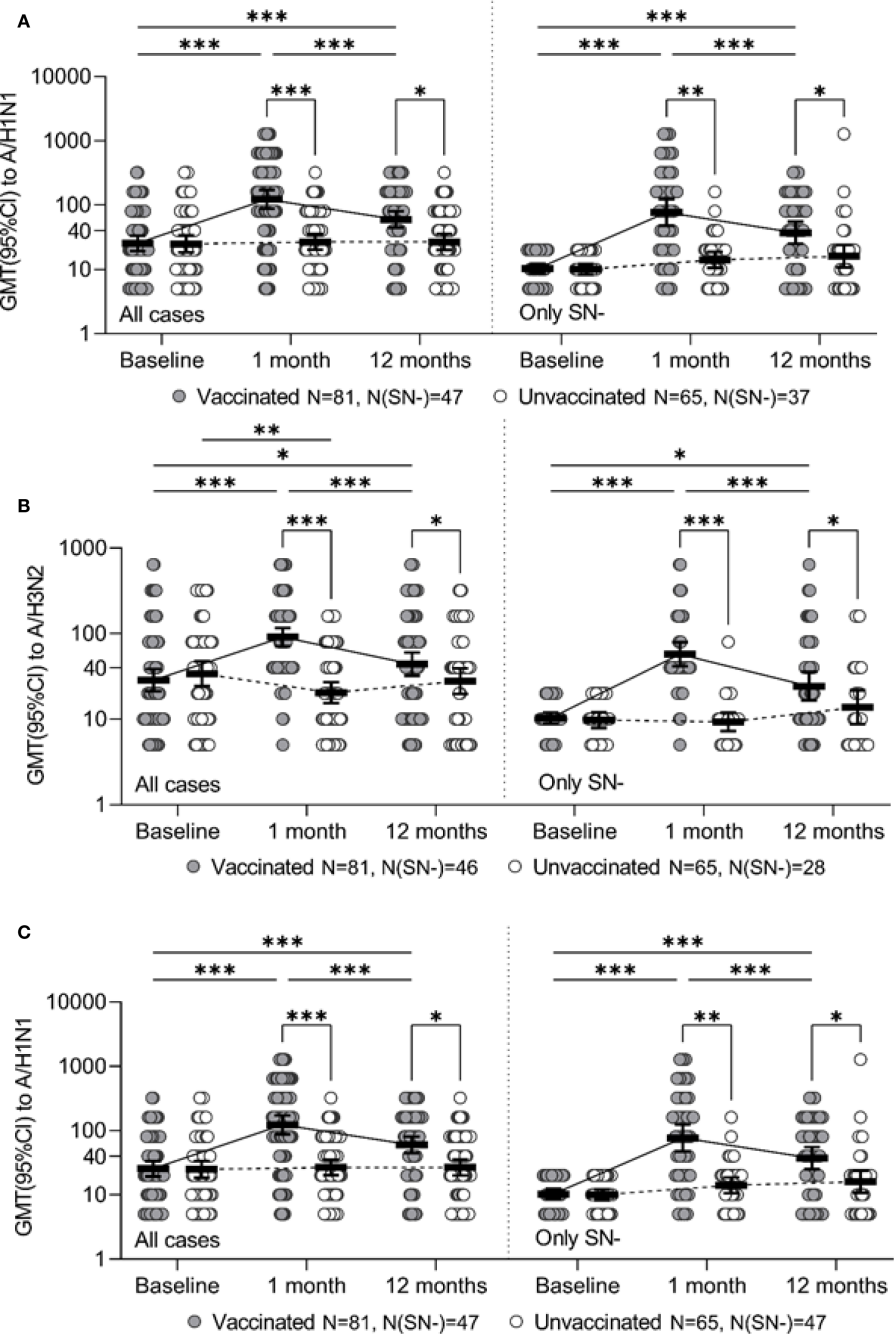
Ab level to the specified strain for the entire study period for all study participants (all cases) and for initially seronegative (only SN-), individual values, geometric mean value and its 95% confidence interval (GMT and 95% CI) are given. Panels **A**, **B**, and **C** represent strains A/H3N2, A/H1N1, and B/Yamagata, respectively. * - p ≤0.05, ** - p <0.01, *** - p <0.001, the corresponding contrasts were constructed within the framework of a robust linear model of mixed effects with the Benjamini-Hochberg correction for multiple comparisons.

Initially comparable in the study groups, the level of Ab to the A/H1N1 strain one month after vaccination statistically significantly increased in the vaccinated group (p <0.001 in each of the seasons and in total for two seasons), while antibody levels in the control group remained unchanged. As a result, one month after the study began, GMT of Ab in the vaccinated group was significantly higher than in the control group: 133.5 [83.8-212.9] vs. 33.1 [21.2-51.6] in the 2014–2015 season (p <0.001), 109.8 [66.3-181.9] vs. 21.3 [15.3-29.8] (p = 0.004) in the 2015–2016 season and 122.7 [87.7-171.8] vs. 26.7 [20.2-35.2] total for both seasons (p <0.001) (**Table 4**). Changes of Аb level to the A/H1N1 (**Table 5**) over time depending on the vaccine status for each analyzed season, in initially seronegative patients are discussed in the next section. The increase in GMT of Ab to the A/H1N1 strain one month post-vaccination relative to baseline (GMFR or seroconversion factor) was 6.2 [3.9-9.7] in the 2014–2015 season, 3.3 [2.2-4.8] in the 2015–2016 season and 4.7 [3.5-6.4] total for both seasons (**Table 6**, all cases). Twelve months after vaccination, Ab to the A/H1N1 strain in the vaccinated group declined from the one-month post-vaccination levels but remained significantly higher than baseline (p <0.001 for the 2014–2015 season, p = 0.009 for the 2015–2016 season and p <0.001 both seasons combined) and was higher than in the control group: 64.8 [43.6-96.2] vs. 26.8 [17.6-40.9] (p = 0.001) in the 2014–2015 season, 53.8 [33.9-85.5] vs. 26.5 [17.6-40.0] (p = 0.04) in the 2015–2016 season and 59.8 [44.5-80.3] vs. 26.7 [20.0-35.5] (p = 0.04) both seasons combined (**Figure 1A**). Interestingly, individuals vaccinated in the 2014–2015 season had significantly higher antibody levels one year post-vaccination compared to those vaccinated in 2015–2016 (p = 0.01). The GMT fold rise relative to baseline was in the 2014–2015 season - 3.0 [2.0-4.4] (p <0.001 vs. control), in the 2015–2016 season - 1.6 [1.1-2.3] (p = 0.05 between seasons, p = 0.37 vs. control), total for both seasons the GMT was 2.3 [1.7-3.0] (p <0.001 vs. control).

**Table 4:** Changes of Аb level over time depending on the vaccine status for each analyzed season and in total, all patients are included in the analysis.

Season	Groups of the study	GMT [95% CI] at control points			P of changes^1^		
		Baseline	1 month	12 months	P^0-1^	P^1-12^	P^0-12^
Strain A (H1N1): 2014–2015 season - A/Victoria/361/2011, 2015–2016 season - A/Switzerland/9715293/2013							
2014 -2015	Vaccinated (N = 46)	21.6 [14.7-31.7]	133.5 [83.8-212.9]	64.8 [43.6-96.2]	p<0.001	p<0.001	p<0.001
	Control group (N = 33)	32.4 [20.0-52.5]	33.1 [21.2-51.6]	26.8 [17.6-40.9]	p=0.96	p = 0.60	p = 0.60
	p of between groups^1^	p=0.57	p<0.001	p = 0.001	–		
2015 -2016	Vaccinated (N = 35)	31.5 [21.4-46.5]	109.8 [66.3-181.9]	53.8 [33.9-85.5]	p<0.001	p<0.001	p = 0.009
	Control group (N = 32)	18.7 [13.0-27.0]	21.3 [15.3-29.8]	26.5 [17.6-40.0]	p=0.65	p=0.55	p=0.17
	p between groups	p = 0.84	p = 0.004	p = 0.04	–		
Total	Vaccinated (N = 81)	25.4 [19.3-33.4]	122.7 [87.7-171.8]	59.8 [44.5-80.3]	p<0.001	p<0.001	p<0.001
	Control group (N = 65)	24.8 [18.3-33.5]	26.7 [20.2-35.2]	26.7 [20.0-35.5]	p=0.74	p = 0.92	p=0.65
	p between groups	p = 0.61	p<0.001	p = 0.04	–		
p season	Vaccinated	p = 0.92	p = 0.09	p = 0.01	–		
	Control group	p = 0.60	p=0.80	p = 0.60			
Strain A (H3N2): 2014–2015 season - A/Victoria/361/2011, 2015–2016 season - A/Switzerland/9715293/2013							
2014 -2015	Vaccinated (N = 46)	52.5 [34.2-80.5]	152.9 [108.0-216.6]	95.9 [66.9-137.3]	p<0.001	0.02	0.001
	Control group (N = 33)	66.2 [40.6-108.0]	41.7 [27.9-62.4]	53.7 [33.1-87.1]	0.03	0.19	0.42
	p of between groups^1^	p = 0.93	p<0.001	p = 0.005	–		
2015 -2016	Vaccinated (N = 35)	12.9 [10.3-16.2]	46.0 [37.7-56.0]	15.8 [11.4-21.7]	<0.001	<0.001	0.67
	Control group (N = 32)	17.2 [12.6-23.4]	9.8 [8.1-11.8]	14.1 [9.7-20.6]	0.01	0.26	0.19
	p between groups	p = 0.97	p<0.001	p = 0.19	–		
Total	Vaccinated (N = 81)	28.7 [21.2-38.7]	91.0 [70.9-116.7]	44.0 [32.1-60.1]	<0.001	<0.001	0.01
	Control group (N = 65)	34.1 [24.5-47.4]	20.4 [15.4-27.1]	27.8 [19.8-39.2]	0.001	0.09	0.14
	p between groups	p = 0.93	p<0.001	p = 0.01	–		
p season	Vaccinated	p = 0.003	p = 0.04	p<0.001	–		
	Control group	p = 0.004	p = 0.002	p = 0.001			
Strain B/Yamagata linage: 2014–2015 season - B/Massachusetts/2/2012, 2014–2015 season - B/Phuket/3073/2013							
2014 -2015	Vaccinated (N = 46)	16.4 [12.1-22.4]	53.3 [37.8-75.1]	27.4 [20.1-37.5]	p<0.001	p<0.001	p = 0.003
	Control group (N = 33)	11.1 [7.5-16.4]	9.6 [6.9-13.3]	7.9 [6.0-10.5]	0.51	0.51	0.20
	p of between groups^1^	p = 0.23	p<0.001	p<0.001	–		
2015 -2016	Vaccinated (N = 35)	22.5 [15.5-32.8]	91.9 [65.7-128.6]	44.2 [34.3-56.9]	<0.001	0.003	0.001
	Control group (N = 32)	17.6 [12.3-25.1]	14.8 [10.6-20.7]	15.8 [12.3-20.3]	0.50	0.69	0.69
	p between groups	p=0.57	p<0.001	p<0.001	–		
Total	Vaccinated (N = 81)	18.7 [14.7-23.7]	66.7 [52.1-85.4]	33.6 [27.2-41.6]	p<0.001	p<0.001	p<0.001
	Control group (N = 65)	13.9 [10.7-18.1]	11.9 [9.4-15.0]	11.1 [9.1-13.6]	0.32	0.81	0.23
	p between groups	p = 0.25	p<0.001	p<0.001	–		

^1^ – a posteriori comparisons were made by constructing the corresponding contrasts within the framework of a robust linear model of mixed effects (**Table 2**), where p^0–1^ is the baseline level and the level after 1 month, p^0–1^ is the level after 1 month and after 12 months, p^0-1^ -baseline level and level after 12 months. For all comparisons within the same strain, the Benjaminii-Hochberg correction for multiple comparisons was applied

**Table 5:** Changes of Аb level over time depending on the vaccine status for each analyzed season, only initially seronegative patients are included in the analysis.

Season	Groups of the study	GMT [95%CI] at control points			P of changes^1^		
		Baseline	1 month	12 months	P^0-1^	P^1-12^	P^0-12^
Strain A (H1N1): 2014–2015 season - A/Victoria/361/2011, 2015–2016 season - A/Switzerland/9715293/2013							
2014 -2015	Vaccinated (N = 32)	10.2 [8.2-12.7]	97.2 [52.3-180.6]	47.6 [29.1-77.8]	p <0.001	p<0.001	p <0.001
	Control group (N = 16)	10.0 [7.0-14.3]	11.4 [7.6-17.2]	11.4 [7.4-17.5]	p=0.78	p=0.96	p = 0.86
	p of between groups^1^	p=0.38	p<0.001	p = 0.02	–		
2015 -2016	Vaccinated (N = 15)	10.5 [7.5-14.7]	46.0 [21.3-99.3]	21.9 [11.3-42.6]	p<0.001	p = 0.02	p = 0.02
	Control group (N = 21)	10.0 [8.0-12.5]	16.4 [10.9-24.8]	20.7 [11.0-39.0]	p = 0.08	p=0.96	p = 0.05
	p between groups	p = 0.33	p = 0.04	p = 0.58	–		
Total	Vaccinated (N = 47)	10.3 [8.6-12.3]	76.5 [47.2-124.0]	37.2 [25.0-55.2]	p<0.001	p<0.001	p<0.001
	Control group (N = 37)	10.0 [8.3-12.1]	14.0 [10.5-18.7]	16.0 [10.7-23.8]	p=0.17	p=0.96	p = 0.18
	p between groups	p = 0.23	p = 0.003	p = 0.04	–		
p season	Vaccinated	p=0.96	p = 0.02	p = 0.03	–		
	Control group	p = 0.94	p=0.38	p = 0.33			
Strain A/H3N2: 2014–2015 season - A/Victoria/361/2011, 2015–2016 season - A/Switzerland/9715293/2013							
2014 -2015	Vaccinated (N = 15)	9.1 [6.6-12.6]	91.9 [37.5-225.3]	57.9 [25.5-131.3]	<0.001	0.08	<0.001
	Control group (N = 6)	7.1 [3.8-13.0]	8.9 [2.8-28.6]	10.0 [2.3-42.8]	0.68	1.00	0.68
	p between groups	p = 0.39	p<0.001	p <0.001	–		
2015 -2016	Vaccinated (N = 31)	10.9 [9.2-13.0]	45.7 [36.6-57.1]	16.0 [11.2-22.8]	<0.001	<0.001	0.38
	Control group (N = 22)	10.7 [8.5-13.4]	9.4 [7.6-11.6]	15.1 [9.1-24.8]	0.68	0.34	0.63
	p between groups	p = 0.91	p<0.001	p=0.77	–		
Total	Vaccinated (N = 46)	10.3 [8.9-12.0]	57.4 [41.7-79.2]	24.3 [16.6-35.8]	<0.001	<0.001	0.02
	Control group (N = 28)	9.3 [7.5-11.5]	9.3 [7.3-11.9]	13.8 [8.8-21.7]	0.88	0.68	0.62
	p between groups	p = 0.47	p<0.001	p = 0.04	–		
p season	Vaccinated	p = 0.63	p<0.001	p = <0.001	–		
	Control group	p = 0.19	p = 0.63	p = 0.23			
Strain B/Yamagata linage: 2014–2015 season - B/Massachusetts/2/2012, 2014–2015 season - B/Phuket/3073/2013							
2014 -2015	Vaccinated (N = 30)	8.7 [7.0-10.8]	40.9 [26.2-64.1]	19.1 [13.0-28.0]	p<0.001	p<0.001	p<0.001
	Control group (N = 27)	7.0 [5.7-8.6]	7.3 [5.6-9.6]	6.0 [5.2-6.9]	p = 0.95	p = 0.58	p = 0.58
	p between groups	p = 0.10	p<0.001	p<0.001	–		
2015 -2016	Vaccinated (N = 17)	8.5 [6.2-11.7]	73.7 [40.4-134.7]	31.3 [23.0-42.6]	p<0.001	p<0.001	p<0.001
	Control group (N = 20)	9.3 [7.1-12.3]	10.4 [7.4-14.6]	14.6 [10.8-19.9]	p = 0.84	p = 0.19	p = 0.11
	p between groups	p = 0.86	p<0.001	p<0.001	–		
Total	Vaccinated (N = 47)	8.6 [7.2-10.3]	50.7 [35.5-72.3]	22.8 [17.4-29.9]	p<0.001	p<0.001	p<0.001
	Control group (N = 47)	7.9 [6.7-9.3]	8.5 [6.9-10.5]	8.8 [7.2-10.7]	p=0.89	p = 0.58	p=0.49
	p between groups	p = 0.26	p<0.001	p<0.001	–		

^1^ – a posteriori comparisons were made by constructing the corresponding contrasts within the framework of a robust linear model of mixed effects (**Table 2**), where p^0–1^ is the baseline level and the level after 1 month, p^0–1^ is the level after 1 month and after 12 months, p^0–1^ is the baseline level and the level after 12 months. For all comparisons within the same strain, the Benjaminii-Hochberg correction for multiple comparisons was applied

**Table 6:** Seroconversion factor 1 month and 12 months after vaccination/start of observation.

Strain/season		1 month after vaccination			12 months after vaccination		
		Vaccinated	Unvaccinated	Between groups	Vac.	Unvac.	Between groups^1^
		GMFR [95%CI]	GMFR [95%CI]		GMFR [95%CI]	GMFR [95%CI]	
All cases							
A/H1N1	Season 2014-2015	6.2 [3.9-9.7]	1.0 [0.8-1.3]	p<0.001	3.0 [2.0-4.4]	0.8 [0.6-1.2]	p<0.001
	Season 2015-2016	3.3 [2.2-4.8]	1.1 [0.8-1.6]	p<0.001	1.6 [1.1-2.3]	1.4 [1.0-2.0]	p=0.37
	Total	4.7 [3.5-6.4]	1.1 [0.9-1.3]	p<0.001	2.3 [1.7-3.0]	1.1 [0.8-1.5]	p = 0.001
	Between seasons^1^	p = 0.08	p = 0.26		p = 0.05	p = 0.09	
A/H3N2	Season 2014-2015	2.9 [1.9-4.4]	0.6 [0.4-0.9]	p<0.001	1.8 [1.1-3.0]	0.8 [0.5-1.2]	p = 0.02
	Season 2015-2016	3.6 [2.8-4.6]	0.6 [0.4-0.8]	p<0.001	1.2 [0.8-1.8]	0.8 [0.5-1.4]	p = 0.19
	Total	3.2 [2.5-4.1]	0.6 [0.5-0.8]	p<0.001	1.5 [1.1-2.1]	0.8 [0.6-1.1]	p = 0.02
	Between seasons	p = 0.09	p = 0.82	–	p = 0.19	p = 0.68	–
B	Season 2014-2015	3.2 [2.2-4.7]	0.9 [0.6-1.2]	p<0.001	1.7 [1.2-2.2]	0.7 [0.6-0.9]	0.008
	Season 2015-2016	4.1 [2.5-6.6]	0.8 [0.6-1.2]	p<0.001	2.0 [1.4-2.8]	0.9 [0.6-1.4]	0.01
	Total	3.6 [2.7-4.8]	0.9 [0.7-1.1]	p<0.001	1.8 [1.4-2.2]	0.8 [0.6-1.0]	<0.001
	Between seasons	p = 0.44	p = 0.73	–	0.54	0.60	
Only seronegative (SN-)							
A/H1N1	Season 2014-2015	9.5 [5.4-16.8]	1.1 [0.8-1.6]	p<0.001	4.7 [2.9-7.4]	1.1 [0.7-1.8]	0.001
	Season 2015-2016	4.4 [2.3-8.4]	1.6 [1.2-2.3]	p = 0.02	2.1 [1.2-3.6]	2.1 [1.1-3.9]	0.68
	Total	7.4 [4.8-11.5]	1.4 [1.1-1.8]	p<0.001	3.6 [2.5-5.2]	1.6 [1.1-2.4]	0.003
	Between seasons	0.05	0.07	–	0.04	0.18	
A/H3N2	Season 2014-2015	10.1 [4.2-24.0]	1.4 [0.6-3.7]	0.02	6.4 [2.6-15.3]	1.6 [0.5-5.2]	0.05
	Season 2015-2016	4.2 [3.4-5.2]	0.9 [0.7-1.2]	<0.001	1.5 [1.0-2.2]	1.4 [0.8-2.5]	0.73
	Total	5.6 [4.0-7.7]	1.0 [0.7-1.3]	<0.001	2.3 [1.5-3.5]	1.5 [0.9-2.3]	0.20
	Between seasons	0.04	0.19	–	0.005	0.73	
B	Season 2014-2015	4.7 [2.9-7.5]	1.1 [0.8-1.4]	p<0.001	2.2 [1.5-3.2]	0.9 [0.7-1.0]	p<0.001
	Season 2015-2016	8.7 [4.1-18.3]	1.1 [0.7-1.7]	p<0.001	3.7 [2.3-5.8]	1.6 [1.0-2.5]	p = 0.02
	Total	5.9 [4.0-8.7]	1.1 [0.9-1.3]	p<0.001	2.6 [2.0-3.5]	1.1 [0.9-1.4]	p<0.001
	Between seasons	p = 0.23	p = 0.66	–	p = 0.08	p = 0.07	

^1^ – Mann-Whitney test with Benjaminii-Hochberg correction was used for multiple testing

### Seronegative participants

A robust mixed-effects model for changes of Ab to the A/H1N1 strain in initially seronegative participants is presented in **Table 3** (SN- only).The analysis of the constructed model revealed that the level of Ab to the A/H1N1 strain in initially seronegative patients, taking into account the correction for interfering factors, were significantly dependent on vaccination status (p < 0.001 at both one month and one year post-vaccination). Additionally, the changes of Ab over time in the group of vaccinated children both one month and one year after the vaccination depended on the season (p = 0.007 and p = 0.006, respectively). In initially seronegative patients, one-month post-vaccination, the geometric mean fold rise (GMFR or seroconversion factor) was: 9.5 [5.4-16.8] times in the 2014–2015 season and 4.4 [2.3 -8.4] times in the 2015–2016 season (p = 0.05 between seasons) - **Table 6** (SN- only). In both seasons, as well as across the entire study period, the increase in Ab one month after vaccination relative to baseline was statistically significant (p <0.001 in each case), the values of the indicators in the vaccinated group one month after the start of the study were significantly higher than in the control group: 97.2 [52.3-180.6] vs. 11.4 [7.6-17.2] in the 2014–2015 season (p <0.001), 46.0 [21.3-99.3] vs. 16.4 [10.9-24.8] in the 2015–2016 season (p = 0.004) and 76.5 [47.2-124.0] vs. 14.0 [10.5-18.7] in total for two seasons (p = 0.003) - **Table 5**. Twelve months post-vaccination, the antibodies against the A/H1N1 strain in the initially seronegative patients declined from the one-month post-vaccination peak but remained significantly above baseline (p <0.001 in the 2014–2015 season, p = 0.02 in the 2015–2016 season and p <0.001 for both seasons combined) - **Figure 1A**). Notably, in the control group there was the increase during the 2015–2016 season in GMT of Ab 12 months after the start of the study (from 10.0 [8.0-12.5] to 20.7 [11.0-39.0], p = 0.05).

Thus, twelve months after the study began, the GMT of Ab in the vaccinated group remained significantly higher than in the control group: in the 2014–2015 season (47.6 [29.1-77.8] vs. 11.4 [7.4-17.5], p = 0.02), comparable to the reference values in the 2015–2016 season (21.9 [11.3-42.6] and 20.7 [11.0 -39.0], respectively, p = 0.58) and total for both seasons (37.2 [25.0-55.2] vs. 16.0 [10.7-23.8], p = 0.04).

Among initially seronegative patients, the seroconversion factor (GMFR, or the fold increase in Ab) 12 months post-vaccination was 4.7 [2.9-7.4] in the 2014–2015 season, 2.1 [1.2- 3.6] in the 2015–2016 season (p = 0.05 between seasons) and 3.6 [2.5-5.2] total for both seasons.

### Antibodies to A/H3N2 strain

Analysis of the post-vaccination immune response to the A/H3N2 strain, adjusted for confounding factors, showed that the level of antibodies to A/H3N2 after vaccination differed statistically significantly one month after vaccination (p <0.001) and one year later (p = 0.003). The dependence of the Ab level on the vaccination season (p = 0.002) and on the initial level of Ab (p <0.001) was also revealed (**Table 3**, all cases). In the 2014–2015 season, initial antibody levels were significantly higher compared to the 2015–2016 season in both study groups, and this difference remained across all subsequent time points (**Table 4**).

One month after vaccination, there was a significant increase in GMT of Ab to the A/H3N2 strain compared to baseline both in overall (p < 0.001) and within each season (p <0.001 - season 2014 - 2015, p <0.001 - season 2015 - 2016), while in the control group one month after the start of the study there was a slight decrease in the Ab level to the A/H3N2 strain compared to baseline (**Figure 1B**). The seroconversion factor (GMFR)—the fold increase in GMT one-month post-vaccination—was: 3.6 [2.8-4.6] in the 2014–2015 season, 2.9 [1.9-4, 4] in the 2015–2016 season and 3.2 [2.5-4.1] overall in both seasons - **Table 6** (all cases). As a result of the described changes, one month after vaccination/start of observation, the GMT of Ab antibodies to the A/H3N2 strain in the group of vaccinated persons were significantly higher than in the non-vaccinated group: 152.9 [108.0-216.6] versus 41.7 [27.9-62.4] in the 2014–2015 season (p <0.001), 46.0 [37.7-56.0] versus 9.8 [8.1-11.8] in the 2015–2016 season (p <0.001) and 91.0 [70.9-116.7] versus 20.4 [15.4-27.1] overall (both seasons) (p <0.001).

Twelve months after vaccination, GMT of Ab to the A/H3N2 strain in the vaccinated group showed a statistically significant decline compared to the one-month level (p = 0.02 - season 2014-2015, p <0.001 - season 2015-2016, p <0.001 - overall (both seasons). However, relative to baseline, antibody levels remained elevated in the 2014–2015 season (p = 0.001) but were comparable to the baseline in the 2015–2016 season (p = 0.67). Compared to the control group, the Ab level to A/H3N2 strain 12 months after vaccination was statistically significantly higher in the 2014–2015 season (95.9 [66.9-137.3] vs. 53.7 [33.1-87.1], p = 0.005) and comparable in the 2015–2016 season (15.8 [11.4-21.7] and 14.1 [9.7-20.6], respectively, p = 0.19). Overall (both seasons) 12 months after vaccination, the level of antibodies to the A/H3N2 strain was 44.0 [32.1-60.1], which is higher than the initial level (28.7 [21.2-38.7], p = 0.01) and higher than in the control group (27.8 [19.8-39.2], p = 0.01).

Twelve months post-vaccination, the seroconversion factor (GMFR) relative to baseline was 1.8 [1.1-3.0] in the 2014–2015 season (p = 0.02 compared to the control group), 1.2 [0.8-1.8] in the 2015–2016 season (no significant difference from the control group, p = 0.19), and overall (both seasons): 1.5 [1.1–2.1] vs. 0.8 [0.6–1.1] in the control group (p = 0.02).

### Seronegative participants

The model describing the formation of post-vaccination immunity (changes of the Ab level over time) to the A/H3N2 strain in the initially seronegative participants is presented in **Table 3** (only SN-). It was revealed that the Ab level to the A/H3N2 strain in initially seronegative patients depended mainly on vaccination (p <0.001 - one month, p = 0.002 - twelve months post-vaccination). It’s also worth mentioning that the changes of Ab over time in the group of vaccinated children one year after the vaccination depended on the season (p = 0.008). Initially comparable GMT of Ab to the A/H3N2 strain between the study groups and seasons one month after vaccination significantly increases in the vaccinated group (p <0.001 - in the 2014–2015 season, p <0.001 – in the 2015–2016 season and p <0.001 total for two seasons) - **Table 5**. It is worth noting that this increase (seroconversion factor) was more intense in the 2014–2015 season compared to the 2015–2016 season (10.1 [4.2-24.0] vs. 4.2 [3.4-5, 2], p = 0.04). The monthly seroconversion factor for the two seasons was 5.6 [4.0-7.7] - **Table 6** (only SN-).

Thus, 1 month after vaccination/study initiation, the GMT of antibodies to the A/H3N2 strain in participants without an initial protective level was significantly higher in the group of vaccinated participants, compared to the control group: 91.9 [37.5-225.3] vs. 8.9 [2.8-28.6] in the 2014–2015 season (p <0.001), 45.7 [36.6-57.1] vs. 9.4 [7.6-11.6] in the 2015–2016 season (p <0.001) and 57.4 [41.7-79.2] vs. 9.3 [7.3-11.9] across both seasons combined (p <0.001) - **Figure 1B**).

Changes of the Ab levels over time to the A/H3N2 strain in initially seronegative patients 12 months after vaccination depended on the study season. In the 2014–2015 season, antibody levels in the vaccinated group remained high (with no significant changes compared to the one-month level, p = 0.08), exceeding baseline values (p < 0.001) and remaining higher than the GMT of Ab to the A/H3N2 strain in the unvaccinated group (p < 0.001). In contrast, in the 2015–2016 season, the GMT of Ab against the A/H3N2 strain declined significantly 12 months after vaccination compared to the one-month level (p < 0.001), returning to baseline values (p = 0.38) and becoming comparable to levels in the unvaccinated group (p = 0.77). Overall, across both seasons, 12 months after vaccination, the level of Ab to the A/H3N2 strain in the initially seronegative study participants was 24.3 [16.6-35.8], which was higher than the initial level (10.3 [8.9-12.0], p = 0.02) and higher than in the control group (12 months after the start of the study - 13.8 [8.8-21.7], p = 0.04).

The seroconversion factor to the A/H3N2 strain in initially seronegative patients 12 months after vaccination, relative to the initial level, was 6.4 [2.6-15.3] in the 2014–2015 season and 1.5 [1.0-2.2] in the 2015–2016 season, with statistically significant differences between seasons (p = 0.005). The overall seroconversion factor (GMFR) across both seasons 12 months after vaccination, relative to the initial level, was 2.3 [1.5–3.5], with no statistically significant differences compared to the unvaccinated group (p = 0.20).

### Antibodies to B strain

The model describing changes in Ab levels over time against strain B is presented in **Table 3** (all cases). Analysis of the constructed model showed that the post-vaccination immune response depended on the patient’s age, with a higher response observed in children over 12 years old (29.2 [24.5-34.8] vs. 18.1 [15.7-20.9], p <0.001), the initial Ab level (p <0.001), and whether the individual was vaccinated in the current season (p <0.001 after 1 month, p = 0.001 after 12 months).

One month after vaccination, the GMT of Ab against the B strain increased significantly in the vaccinated group compared to baseline (p <0.001 in each season and total across both seasons) - **Table 4**. Twelve months after vaccination, there was a decline in the Ab levels to B strain compared to the one-month level (p <0.001 for the 2014–2015 season, p = 0.003 for the 2015–2016 season and p <0.001 for both seasons combined). However, GMT values remained above baseline (p = 0.003 for the 2014–2015 season, p = 0.001 for the 2015–2016 season, p <0.001 for both seasons combined). In the control group, no statistically significant changes in GMT of Ab to B strain were observed throughout the study period. As a result, one month after vaccination/start of the study, the GMT of Ab to strain B was significantly higher in the vaccinated group compared to the control group (53.3 [37.8-75.1] vs. 9.6 [6.9-13.3] (p <0.001) in the 2014–2015 season, 91.9 [65.7-128.6] vs. 14.8 [10.6-20.7] (p <0.001) in the 2015–2016 season and 66.7 [52.1-85.4] vs. 11.9 [9.4-15.0] (p <0.001) both seasons combined). Twelve months after vaccination, GMT antibody levels in the vaccinated group remained significantly higher than in the control group (27.4 [20.1-37.5] versus 7.9 [6.0-10.5] (p <0.001) in the 2014–2015 season, 44.2 [34.3-56.9] vs. 15.8 [12.3-20.3] (p <0.001) in the 2015–2016 season and 33.6 [27.2-41.6] vs. 11.1 [9.1-13.6] (p <0.001) in total for two seasons) - **Figure 1C**).

The seroconversion factor (GMFR) in the vaccinated group one month after vaccination compared to the baseline level was 3.2 [2.2-4.7] in the 2014–2015 season (p <0.001 vs. control), 4.1 [2.5-6.6] in the 2015–2016 season (p <0.001) and 3.6 [2.7-4.8] for both seasons combined (p <0.001); twelve months after vaccination - 1.7 [1.2-2.2] in the 2014–2015 season (p = 0.008 vs. control), 2.0 [1.4-2.8] in the 2015–2016 season (p = 0.01) and 1.8 [1.4-2.2] for both seasons combined (p <0.001) – see **Table 6** (all cases).

### Seronegative participants

The analysis of the changes in Ab levels over time to strain B in the initially seronegative study participants is given in **Table 3** (only SN-). It was revealed that the level of antibodies to strain B in initially seronegative patients depended only on the fact of vaccination (p <0.001 after 1 month, p = 0.001 after 12 months). The changes of the Ab level over time to strain B in initially seronegative participants is characterized by a statistically significant increase one month after vaccination compared to baseline (p <0.001 for both seasons and in total for two seasons) with a subsequent decrease after 12 months (p <0.001 for both seasons and in total across two seasons), while the GMT of Ab to B strain and one year after vaccination remained above the baseline (p <0.001 for both seasons and in total for two seasons) - **Table 5**. When compared to the control group, GMT of Ab levels to B strain in initially seronegative patients in the vaccinated group were significantly higher both one month (40.9 [26.2-64.1] vs. 7.3 [5.6- 9.6] in the 2014–2015 season (p <0.001), 73.7 [40.4-134.7] vs. 10.4 [7.4-14.6] in the 2015–2016 season (p <0.001) and 50.7 [35.5-72.3] vs. 8.5 [6.9-10.5] total for two seasons (p <0.001)) and one year post-vaccination (19.1 [13.0 -28.0] vs. 6.0 [5.2-6.9] in the 2014–2015 season (p <0.001), 31.3 [23.0-42.6] vs. 14.6 [10.8- 19.9] in the 2015–2016 season (p <0.001) and 22.8 [17.4-29.9] vs. 8.8 [7.2-10.7] total for two seasons (p <0.001)) - **Figure 1C**).

Among initially seronegative patients, the seroconversion factor (GMFR, or the fold increase in Ab) one month after vaccination was 4.7 [2.9-7.5] in the 2014–2015 season (p <0.001 compared to the control), 8.7 [4.1-18.3] in the 2015–2016 season (p <0.001), and 5.9 [4.0-8.7] total for two seasons (p <0.001); 12 months post-vaccination was 2.2 [1.5-3.2] in the 2014–2015 season (p <0.001), 3.7 [2.3-5.8] in the 2015–2016 season (p = 0.02), and 2.6 [2.0-3.5] total for two seasons (p <0.001) - **Tables 6** and **7** (only SN-).

**Table 7:** Seroconversion level and seroconversion factor after 1 month and 12 months after vaccination/start of observation relative to the baseline level.

Strain/ season		Seroconversion rate - % [95%CI]						Seroconversion factor - % [95% CI]					
		1 month			12 months			1 month			12 months		
		Vac.	Unvac.	P^1^	Vac.	Unvac.	P	Vac.	Unvac.	P^2^	Vac.	Unvac.	P
All cases													
A/H1N1	Season 2014-2015	63% [49-75]	9% [3-24]	p<0.001	50% [36-64]	9% [3-24]	p<0.001	80% [67-89]	52% [35-67]	p = 0.008	76% [62-86]	45% [30-62]	p = 0.01
	Season 2015-2016	49% [33-64]	9% [3-24]	p<0.001	23% [12-39]	22% [11-39]	p = 0.92	80% [64-90]	41% [26-58]	p = 0.003	63% [46-77]	50% [34-66]	p=0.36
	Total	57% [46-67]	9% [4-19]	p<0.001	38% [28-49]	15% [9-26]	p = 0.005	80% [70-87]	46% [35-58]	p<0.001	70% [60-79]	48% [36-60]	p = 0.01
	P season^3^	p = 0.28	p=1.00	–	p = 0.03	p = 0.19	–	p=0.96	p = 0.56	–	p = 0.32	p=0.71	–
A/H3N2	Season 2014-2015	37% [25-51]	3% [1-15]	p<0.001	35% [23-49]	9% [3-24]	p = 0.02	91% [80-97]	73% [56-85]	p = 0.04	87% [74-94]	79% [62-89]	p=0.55
	Season 2015-2016	66% [49-79]	3% [1-16]	p<0.001	20% [10-36]	13% [5-28]	p=0.51	86% [71-94]	0% [0-11]	p<0.001	23% [12-39]	31% [18-49]	p=0.55
	Total	49% [39-60]	3% [1-11]	p<0.001	28% [20-39]	11% [5-21]	p = 0.02	89% [80-94]	37% [26-49]	p<0.001	59% [48-69]	55% [43-67]	p = 0.64
	P season	p = 0.01	p = 0.98	–	p = 0.35	p=0.65	–	p=0.49	p<0.001	–	p<0.001	p<0.001	–
B	Season 2014-2015	41% [28-56]	6% [2-20]	p<0.001	30% [19-45]	0% [0-10]	p<0.001	72% [57-83]	18% [9-34]	p<0.001	54% [40-68]	12% [5-27]	p<0.001
	Season 2015-2016	51% [36-67]	13% [5-28]	p = 0.002	37% [23-54]	22% [11-39]	p=0.21	83% [67-92]	19% [9-35]	p<0.001	63% [46-77]	22% [11-39]	p = 0.001
	Total	46% [35-56]	9% [4-19]	p<0.001	33% [24-44]	11% [5-21]	p<0.001	77% [66-84]	18% [11-30]	p<0.001	58% [47-68]	17% [10-28]	p<0.001
	P season	p=0.38	p=0.38	–	p = 0.53	p=0.007	–	p = 0.31	p = 0.95	–	p = 0.44	p=0.36	–
Only seronegative (SN-)													
A/H1N1	Season 2014-2015	75% [58-87]	19% [7-43]	p<0.001	72% [55-84]	19% [7-43]	p<0.001	72% [55-84]	6% [1-28]	p<0.001	69% [51-82]	6% [1-28]	p<0.001
	Season 2015-2016	60% [36-80]	14% [5-35]	p=0.007	27% [11-52]	24% [11-45]	p=1.00	60% [36-80]	24% [11-45]	p = 0.05	40% [20-64]	29% [14-50]	p = 0.47
	Total	70% [56-81]	16% [8-31]	p<0.001	57% [43-70]	22% [11-37]	p = 0.003	68% [54-80]	16% [8-31]	p<0.001	60% [45-72]	19% [9-34]	p<0.001
	P season	p = 0.40	p=1.00	–	p = 0.005	p=1.00	–	p=0.51	p = 0.19	–	p=0.13	p=0.13	–
A/H3N2	Season 2014-2015	80% [55-93]	17% [3-56]	p = 0.02	60% [36-80]	17% [3-56]	p = 0.05	73% [48-89]	17% [3-56]	p = 0.03	73% [48-89]	17% [3-56]	p = 0.05
	Season 2015-2016	74% [57-86]	5% [1-22]	p<0.001	23% [11-40]	18% [7-39]	p = 0.94	84% [67-93]	0% [0-15]	p<0.001	23% [11-40]	32% [16-53]	p = 0.53
	Total	76% [62-86]	7% [2-23]	p<0.001	35% [23-49]	18% [8-36]	p = 0.25	80% [67-89]	4% [1-18]	p<0.001	39% [26-54]	29% [15-47]	p = 0.35
	P season	p=1.00	p=0.49	–	p = 0.05	p=1.00	–	p = 0.45	p = 0.05	–	p = 0.005	p = 0.53	–
B	Season 2014-2015	57% [39-73]	7% [2-23]	p<0.001	40% [25-58]	0% [0-12]	p<0.001	63% [46-78]	4% [1-18]	p<0.001	40% [25-58]	0% [0-12]	p<0.001
	Season 2015-2016	71% [47-87]	15% [5-36]	p = 0.002	65% [41-83]	35% [18-57]	p = 0.09	71% [47-87]	10% [3-30]	p<0.001	41% [22-64]	20% [8-42]	p = 0.20
	Total	62% [47-74]	11% [5-23]	p<0.001	49% [35-63]	15% [7-28]	p<0.001	66% [52-78]	6% [2-17]	p<0.001	40% [28-55]	9% [3-20]	p<0.001
	P season	p = 0.44	p = 0.64	–	p = 0.10	p = 0.002	–	p = 0.75	p = 0.47	–	p=1.00	p = 0.02	–

^1^ - χ^2^ test was used (Fisher in case of presence of cells with expected frequencies ≤5%) with Benjaminii-Hochberg correction for multiple comparisons

* - p ≤0.05, ** - p <0.01, *** - p <0.001, the corresponding contrasts were constructed within the framework of a robust linear model of mixed effects with the Benjamini-Hochberg correction for multiple comparisons

### Compliance of the vaccine with the CPMP/BWP/214/96 criteria

An analysis of the vaccine’s compliance with the CPMP/BWP/214/96 criteria showed that the level of seroprotection one month after vaccination in total for the two seasons 2014–2015 and 2015–2016 was 80 [70–87]% to A/H1N1, 89 [80–94]% to A/H3N2 and 77 [66-84]% to strain B, which met the criteria (seroprotection level target: >70%) and was significantly higher than the values in the control group (46 [35-58]%, 37 [26-49]% and 18 [11-30]%, respectively, p <0.001 for each strain). A similar pattern (compliance with the criterion and statistically significant excess of the indicator values in the control group) is typical for each season considered - **Table 7** (all cases).

The seroconversion rate also met the CPMP criteria (target: > 40%) and significantly exceeded the values in the control group: 57% [46-67%] vs. 9% [4-19%] for A/H1N1 (p <0.001), 49% [39-60%] vs. 3% [1-11%] for A/H3N2 (p <0.001), and 46% [35-56%] vs. 9% [4-19%] for strain B (p <0.001).

The seroconversion factor for two seasons to the A/H1N1 strain in the vaccinated group was 4.7 [3.5-6.4] vs. 1.1 [0.9-1.3] in the control group (p <0.001), to the A/H3N2 strain - 3.2 [2.5-4.1] vs. 0.6 [0.5-0.8] (p <0.001), for strain B - 3.6 [2.7-4.8] vs. 0.9 [0.7-1.1] (p <0.001), meeting CPMP criteria (which must be at least 2.5-fold) in total and in each of the seasons alone- **Table 6** (all cases).

Also, the level of seroprotection, factor and level of seroconversion were calculated separately for seronegative patients. One month after the vaccination, the level of seroprotection (combined across both seasons) to strains A/H1N1, A/H3N2 and B was 68 [54-80]%, 80 [67-89]% and 66 [52-78]%, respectively, seroconversion rate -70 [56-81]%, 76 [62-86]% and 62 [47-74]%, seroconversion factor-7.4 [4.8-11.5], 5.6 [4, 0-7.7] and 5.9 [4.0-8.7]. Detailed calculations for each season, as well as efficacy indicators 12 months post-vaccination, are presented in **Table 6** and **Table 7**.

**Figure 2:**
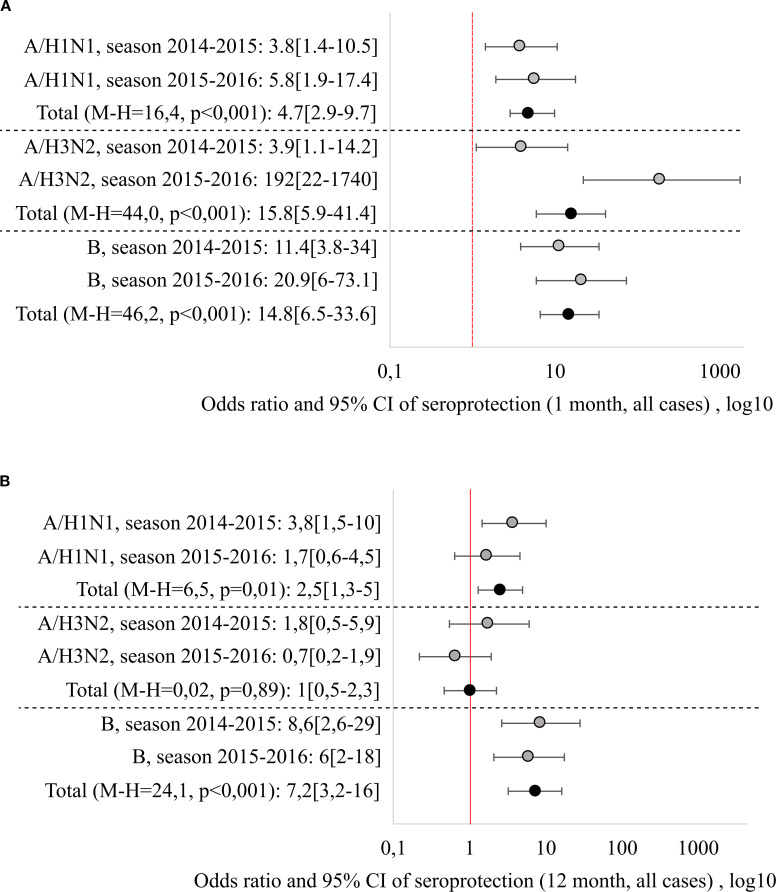
Relative risks of reaching seroprotective level 1 month **(A)** and 12 months **(B)** after vaccination/beginning of observation, generalization by seasons was carried out by the Mantel-Haenszel method, with the calculation of the Cochran-Mantel-Hensel criterion.

It should be noted that there is a fairly strong variability in the performance indicators depending on the season, including the results of comparisons with the control group. In order to eliminate this variability and to generalise the results obtained, odds ratios were calculated, which indicate how many times the chance of achieving a seroprotective level of Ab (above 40) is greater in the vaccinated group than in the unvaccinated group. To aggregate the odds ratios across different epidemiological seasons, the Mantel-Henszel odds ratio was calculated, with the method’s conditions verified using the Breslow-Day test. The relative odds of achieving seroprotection one month after vaccination/study entry and of maintaining a protective Ab level 12 months in the vaccinated group (compared to unvaccinated group) are shown in **Figure 2**.

Regardless of the epidemiological season, vaccination significantly increased the chance of reaching seroprotective Ab level one month after vaccination: the combined odds ratios (Mantel-Henszel method) were: 4.7 [2.9-9.7] for the A/H1N1 strain (χ²MH = 16.4, p < 0.001), 15.8 [5.9-41.4] for the A/H3N2 strain (χ²MH = 44.0, p < 0.001) and 14.8 [6.5-33.6] for strain B (χ²MH = 46.2, p < 0.001).

Another situation was observed when analyzing the chances of maintaining the seroprotective level 12 months after the vaccination. The highest persistence was shown by Ab to B strain, with the odds of maintaining seroprotection 7.2 times higher than in the non-vaccinated group (OR = 7.2 [3.2–16]), independent of the season. Ab resistance to the A/H1N1 strain was season-dependent (it was lower in the 2015–2016 season), the combined Mantel-Henszel odds ratio across seasons was 2.5 [1.3–5] (χ²MH = 6.5, p = 0.01). Ab to the A/H3N2 strain demonstrated the least persistence: one year post-vaccination, the odds of maintaining seroprotection were equivalent to the non-vaccinated group (OR = 1.0 [0.5–2.3], χ²MH = 0.02, p = 0.89).

## Discussion

The increase in the prevalence and incidence of diabetes, including T1D among children and adolescents, is a growing global health problem. This issue has prompted many specialists to seek more effective treatment and disease management strategies. Vaccination of patients with diabetes against influenza has been shown to be both effective and safe. However, the factors influencing influenza vaccination among children have not been studied as comprehensively as those in adults. A recent study conducted in Madrid [35] showed that diabetes was risk condition strongly associated with a higher rate of vaccination adherence (OR = 2.15; 95% CI, 1.74-2.65). Of the vaccinated children with type 1 diabetes, 46.5% were vaccinated with at least one dose, and 45.6% were fully vaccinated. Since children with T1D frequently visit healthcare providers (pediatricians and pediatric endocrinologists), this opens up many opportunities for educating parents and promoting vaccination - not only against influenza, but also against pneumococcal infection and COronaVIrus Disease 2019 (COVID-19), depending on the current epidemiological context (5, 36, 37, 53).

Recent studies have shown that repeated annual influenza vaccinations were associated with a reduced antibody response induced by the vaccine (38), but a higher frequency of vaccine-specific regulatory T (Treg) cells. Logistic regression revealed that previous vaccination was significantly associated with a decrease in the likelihood of achieving seroconversion both for the A/H1N1 strain (adjusted OR = 0.03 (95% CI, 0.01–0.13), and for A/H3N2 (A/H3N2 strain: adjusted OR = 0.09 (95% CI, 0.03–0.30) (39). Thus, when studying the association of seroconversion with the pre-existing T regulatory cells of the vaccine and the ratio of regular T cells (Tconv/T) cells investigated the relationship between HA(H3)-specific T cells and the effectiveness of the vaccine it was found that in one month after vaccination and HA(H3)-specific T cells. Significant changes were observed in those vaccinated who had not previously been vaccinated. Significantly higher seroconversion rates for H1N1 and H3N2 than for those who had pre-vaccination (60% vs. 5% for H1N1; 67% vs. 15% for H3N2). This trend was observed even 12 months after vaccination. Interestingly, compared with those vaccinated without seroconversion, those vaccinated who showed seroconversion (1 month after vaccination) had a significantly lower frequency of pre-existing HA(H3)-specific cells before vaccination (0.000596 vs. 0.000369, n = 0.034) and a higher Tconv/Treg ratio (n = 0.048), but a similar frequency of Tconv. Twelve months after vaccination with vaccines those with seroconversion had significantly higher rates of Tconv/THC ratio, but similar frequencies of HA(H3)-specific Tconv and Thc cells were compared with those without seroconversion. Lower levels of pre-existing HA(H3)-specific Treg and an ably higher Tconv/Treg cell ratio were associated with a higher likelihood of seroconversion caused by seasonal influenza vaccine. The analysis of the relationship between previous vaccination and pre-existing T cells, immunity and post-vaccination seroprotection (HI titer ≥ 1:40) showed that Ab baseline, individuals who received the previous vaccination had significantly higher SPR rates for H1N1 and H3N2 compared to those who did not receive the previous vaccination (H1N1: 79.3% vs. 50%; H3N2: 60.9% vs. 25%). One month after vaccination, both groups demonstrated that they also analyzed similar seroprotection rates for H1N1 and H3N2. The multiplicity of changes in geometric mean HI titers 1 month after vaccination compared to baseline. Interestingly, vaccinations without seroprotection at baseline showed a significantly higher three-fold increase in vaccine-induced H1N1 HI titers (13.97 times) compared to those who had seroprotection at the initial level (1.65 times). Also, then fold to change the H3N2 HI titers 1 month after vaccination was significantly higher in the initially seronegative vaccinated compared with the initially seropositive vaccinated (2.91 folds versus 1.71 folds). Thus in addition, influenza vaccination provides excellent seroprotection, and vaccine-induced seroprotection is associated with a lower level of pre-existing inhibition of HA(H3)-specific Treg cells in vaccinated individuals without previous influenza vaccination.

In contrast to these findings, in our study vaccination against influenza of children with T1D using a trivalent immunoadjuvanted vaccine showed that in initially seropositive patients, GMT of Ab and seroconversion factor to A/H1N1 and A/H3N2 strains twelve months after vaccination are higher compared to initially seronegative patients, regardless of the vaccination season. The results obtained are likely related to the immunoadjuvant azoximer bromide which could be accompanied by significantly less activation of antigen-specific Treg cells, compared with the non-adjuvanted vaccine and reduced the spread of antigen-specific Treg cells upon re-immunization, similar to CpG-adjuvanted peptide vaccines provide heterosubtypic influenza protection probably by inhibiting Treg development and enhancing T cell immunity (40). However, it is necessary to note that this study solely focused on analyzing H3-specific T-cell responses.

In initially seronegative patients with T1D, despite a significant increase in Ab levels in the first month after vaccination, the GMT of Ab to strains A/H1N1 and A/H3N2 was significantly higher in the vaccinated group compared to the control group in the 2014–2015 season, but comparable to the reference values in the 2015–2016 season in the non-vaccinated group at 12 months after the start of the study. It is possible that the formation of specific antibodies in initially seronegative patients is influenced by the seasonal circulation of influenza viruses, which contribute to the boosterization of post-vaccination Ab. An analysis of literary sources devoted to the epidemiological situation with influenza in Russia in the period from 2014 to 2015 showed that the main cause of the disease was influenza B, which accounted for 50.6% of all detected strains. Influenza A(H3N2) viruses accounted for 45% of all isolates, and the 2009 pandemic virus A(H1N1)pdm09 was detected in only 4.35% of clinical materials (41, 42). In the 2014–2015 season, the new strain of the A(H3N2) virus, A/Switzerland/9715293/2013, was the predominant influenza agent in most countries of the Northern Hemisphere. This virus differed from the vaccine strain A/Texas/50/2012 not only in its antigenic properties, but also became the ancestor of a new genetic line in the evolution of influenza. A/Switzerland/9715293/2013 (H3N2) caused a more intense epidemic, during which cases of influenza were recorded even in vaccinated people, as well as fatal outcomes among children and the elderly. High activity of the influenza B virus was observed throughout the season, and it became dominant in almost all countries in April-May 2015. In addition, an extremely low activity of the influenza A(H1N1)pdm09 virus was recorded, but its pathogenic properties were preserved, which led to the development of severe forms of infection, often with a fatal outcome. In the 2014–2015 season, one interesting feature was observed: the properties of the vaccine strains A(H3N2) and B were not completely similar. This led to the replacement of these strains in the WHO recommendations for the 2015–2016 season (43). Thus, the epidemiological situation associated with influenza in the period from 2014 to 2015 in Russia was characterized by an average intensity of morbidity caused by influenza B and A(H3N2) viruses. This probably contributed to additional activation of post-vaccination immunity. It should be noted that in this season, a high level of post-vaccination antibodies to the influenza A(H1N1) pdm09 virus was observed, despite the relatively low incidence. This probably indicates the high immunogenicity of the pandemic strain, and its long-term use in vaccines apparently influenced the formation of population immunity.

In the 2015–2016 flu season, in most countries of the Northern Hemisphere, there was a widespread dominance of influenza A (H1N1) pdm09 virus strains, amounting to more than 90%. This fact was predicted and described in the work of domestic authors after it was included in the recommendations for the composition of vaccines for this season. The high level of morbidity can be explained by the fact that the A(H1N1)pdm09 influenza viruses have not undergone significant antigenic drift since their entry into the human population in 2009. The reference strain A/California/07/09 still remains the main one for the production of influenza vaccines. Given the low level of circulation of this influenza subtype in the current epidemic season, it could be expected that its contribution to the epidemic process would increase in the next season of 2015–2016. These forecasts came true (41).

In Russia in 2015-2016, influenza A (H1N1)pdm09 was predominant - its share in the structure of circulating viruses was 84%. The activity of influenza viruses A (H3N2) and B is significantly lower - 7% and 9%, respectively (44).

Based on the results of studying the antigenic properties of the influenza A (H1N1)pdm09 virus strains, no differences were found in relation to the vaccine virus, but the strains of influenza A (H3N2) and B viruses did not match in composition (45). We do not yet know why, despite the high incidence of influenza A (H1N1)pdm09 and the low incidence of influenza A (H3N2) and B viruses, we found a significant increase in post-vaccination antibodies to the B strain, which persisted 12 months after vaccination. At the same time, the level of antibodies to influenza A (H1N1)pdm09 strains and especially to A (H3N2) was low. It can be assumed that there is some antagonism between the production of post-vaccination antibodies to influenza A (H1N1)pdm09 and circulating strains, which is manifested in the activation of antigen-specific Treg cells (39). The low level of post-vaccination antibodies to the influenza A(H3N2) virus strain is likely due to its variability and frequent substitutions in vaccines, including the 2015–2016 season. This results in low antibody levels after the first year of vaccine administration. The significant increase in antibodies to the B/Phuket/3073/2013 strain, which persists even one year after vaccination despite its substitution in vaccines in the 2015–2016 season, may be due to the fact that mainly Yamagata lineage viruses circulated during the last two epidemic seasons. The same B/Massachusetts/2/2012 strain was used in both vaccines. Therefore, the use of the B/Phuket/3073/2013 strain from the same lineage likely contributed to the development of herd immunity.

A particularly important finding was the decline of post-vaccination Abs to the A/H3N2 strain following vaccination with inactivated influenza vaccines (46, 47). Also it is thought that the previous seasonal vaccination adversely affected the strength of the immune response to A/H1N1 caused by influenza vaccines. As for H1N1, the geometric mean titer at 30 days post‐vaccination was lower in repeated vaccinated participants compared to participants with or without prior vaccination, but the proportion with titers ≥40 was consistent in both groups. Nevertheless, the authors conclude that repeated vaccination provides similar or enhanced protection compared than single vaccination in newly vaccinated individuals (47). Other researchers have shown that residual protection against influenza viruses persists, even when circulating viruses differ antigenically from those included in the previous season’s vaccine (48). In general, vaccination provides immune protection regardless of vaccination history and remains beneficial, particularly for patients who have lost protective antibodies.

One of the most recent meta-analyses, which included 1918 articles on the maintenance of post-vaccination immunity in those vaccinated with standard and adjuvanted influenza vaccines, showed that the use of adjuvants has a significant advantage in the duration of maintenance of post-vaccination immunity, especially in children (49, 50). Adjuvants such as imiquimod, GLA, MF59 and AS03 have been studied and have been shown to increase the antibody response against homologous and heterologous strains of influenza virus compared with nonadjuvanted vaccines (51).

A peculiarity of our study was the use of the vaccine with an immunoadjuvant in children with T1D of different duration of diabetes of the disease during two consecutive seasons. No analogous studies were found in the available literature. The analysis of the results of the evaluation of the immunogenicity of the vaccine in children with T1D showed that regardless of the season of its use, as well as across for both seasons of 2014–2015 and 2015-2016, the level of seroprotection, the level of seroconversion and the seroconversion factor for all 3 strains of the influenza virus in one month after vaccination, met the CPMP criteria CPMP/BWP/214/96. It should be noted that in children with T1D who had not received any previous influenza vaccination (seronegative), seroprotection (which should be at least 70%) one month after vaccination was 68% [54 to 80%] for A/H1N1, 80% [67 to 89%] for A/H3N2, and 66% [52 to 78%] for strain B, for a total of two seasons. The level of seroconversion and the seroconversion factor in these initially seronegative patients with T1D met the CPMP criteria.

Since there was a fairly strong variability in the immunogenicity of the vaccine depending on the season (2014–2015 and 2015-2016), including the difference in the results of the comparison with the control group, the odds ratios of maintaining the seroprotective level 12 months after vaccination were calculated. It was found that Ab to strain B showed the greatest resistance, where the chance of having a protective level 12 months after vaccination was 7.2 [3.2-16] times higher than that of the unvaccinated, and did not depend on the season. Ab resistance to the A/H1N1 strain depended on the season (it was lower in the 2015–2016 season), the combined odds ratio was 2.5 [1.3-5] times (χ2M-H = 6.5, p = 0.01), while Ab to the A/H3N2 strain, one year after vaccination showed the least resistance and the chance of having a protective level of Ab was the same as in unvaccinated ones - 1.0 [0.5-2.3] (χ2M - H = 0.02, p = 0.89).

Another special feature of our study was that we consider the epidemiological context of these seasons. It should not be overlooked that the Ab level against different influenza viruses is influenced not only by vaccination (including prior-year vaccination), but also by exposure to the pathogen. Thus, in the 2014–2015 season in our region compared to 2013, the circulation of influenza B (10 times) and A/H3N2/(2.7 times) viruses increased significantly against the background of the almost disappearance of A/H1N1 (seasonal) and A/H1N1/09 (highly pathogenic), and in the 2015–2016 season the circulation of viruses of influenza etiology decreased by 1.8 times, including influenza B (by 6 times) (52). Therefore, the study of the Ab levels to the A/H1N1 virus accurately reflects post-vaccination immunity.

Thus, the trivalent immunoadjuvanted subunit influenza vaccine, containing a 3-fold reduction in the amount of hemagglutinin for each of the three epidemic influenza virus strains and 500 μg of the water-soluble immunoadjuvant Polyoxidonium per dose, demonstrated a high levels of seroprotection against all strains included in the current season in children with T1D, regardless of prior vaccination history. In contrast to seronegative patients, vaccination of seropositive patients contributed to the maintenance of specific Ab to all three influenza strains for at least one year.

## Conclusions

The administration of a trivalent immunoadjuvanted subunit influenza vaccine in children with T1D results in post-vaccination antibody formation that meets CPMP immunogenicity criteria, regardless of prior vaccination history.

## Data Availability

The original contributions presented in the study are included in the article/supplementary material. Further inquiries can be directed to the corresponding author.
